# Mutational analysis of epidermolysis bullosa in Taiwan by whole-exome sequencing complemented by RNA sequencing: a series of 77 patients

**DOI:** 10.1186/s13023-022-02605-1

**Published:** 2022-12-28

**Authors:** Wei-Ting Tu, Ping-Chen Hou, Peng-Chieh Chen, Wan-Rung Chen, Hsin-Yu Huang, Jing-Yu Wang, Yi-Ting Huang, Yi-Huei Wu, Chun-Lin Su, Yen-An Tang, Hiroaki Iwata, Ken Natsuga, Sheau-Chiou Chao, H. Sunny Sun, Ming-Jer Tang, Julia Yu-Yun Lee, John A. McGrath, Chao-Kai Hsu

**Affiliations:** 1 Department of Dermatology, College of Medicine, National Cheng Kung University Hospital, National Cheng Kung University, 138 Sheng-Li Road, Tainan City, Taiwan; 2 Institute of Clinical Medicine, College of Medicine, National Cheng Kung University, Tainan, Taiwan; 3 School of Medicine, College of Medicine, National Cheng Kung University, Tainan, Taiwan; 4 Department of Biomedical Engineering, National Cheng Kung University, Tainan, Taiwan; 5 International Center for Wound Repair and Regeneration (iWRR), National Cheng Kung University, Tainan, Taiwan; 6 Institute of Molecular Medicine, College of Medicine, National Cheng Kung University, Tainan, Taiwan; 7 Center for Genomic Medicine, Innovation Headquarters, National Cheng Kung University, Tainan, Taiwan; 8 Department of Dermatology, Faculty of Medicine and Graduate School of Medicine, Hokkaido University, Sapporo, Japan; 9 St. John’s Institute of Dermatology, King’s College London (Guy’s Campus), London, UK

**Keywords:** Epidermolysis bullosa, Whole-exome sequencing, RNA sequencing

## Abstract

**Background:**

Epidermolysis bullosa (EB) is a heterogeneous group of hereditary skin diseases characterized by skin fragility. Primary data on Taiwanese population remain scarce.

**Methods:**

We gathered clinical information from EB patients at National Cheng Kung University Hospital from January, 2012, to June, 2021. Diagnostic tests including transmission electron microscopy, immunofluorescence studies, and whole-exome sequencing (WES) were performed. The pathogenicity of novel splice-site mutations was determined through reverse transcriptase-PCR of skin mRNA followed by Sanger and/or RNA sequencing.

**Results:**

Seventy-seven EB patients from 45 families were included: 19 EB simplex, six junctional EB, and 52 dystrophic EB. Pathogenic variants were identified in 37 of 38 families (97.4%), in which WES was used as a first-line tool for mutational analysis; RNA sequencing determined pathogenic variants in the remaining one family. A total of 60 mutations in EB-related genes were identified, including 22 novel mutations. The mutations involved *KRT5*, *KRT14*, *PLEC*, *COL17A1*, *LAMB3*, *LAMA3*, *ITGB4*, and *COL7A1*. Over one-quarter of DEB patients had EB pruriginosa.

**Conclusions:**

The distinct clinical presentation and molecular pathology of EB in Taiwan expand our understanding of this disorder. WES was an effective first-line diagnostic tool for identifying EB-associated variants. RNA sequencing complemented WES when multiple potentially pathogenic splice-site mutations were found.

**Supplementary Information:**

The online version contains supplementary material available at 10.1186/s13023-022-02605-1.

## Introduction

Inherited EB is a group of rare heterogeneous hereditary diseases in which there is skin, and sometimes mucosal, fragility following minor trauma. Classical forms of EB result from mutation in one of 16 genes encoding proteins responsible for maintaining cellular integrity and adhesion of the skin and/or mucosa, while mutations in a further 24 genes may contribute to skin fragility in other non-classical disorders encompassed by the umbrella term EB [1, 2]. In addition to mucocutaneous fragility and scarring, EB also causes numerous other manifestations, including gastrointestinal and urethral strictures, anemia, failure to thrive, muscular dystrophy, and cutaneous malignancies [1].

With the introduction of transmission electron microscopy (TEM), immunofluorescence microscopy (IF) studies, and mutational analysis to EB research and diagnostics, our understanding of the disease has increased tremendously over the last 50 years. Classical forms of EB are classified as EB simplex (EBS), junctional EB (JEB), dystrophic EB (DEB), or Kindler EB, based on the level of the cleavage plane: above, within, or beneath the basement membrane zone. Patients can be further categorized into one of the 35 subtypes, according to the 2020 EB consensus reclassification, based on clinical, pathologic, and genetic findings [2].

Previously, reports on the clinical phenotype and molecular pathology of EB have focused mainly on European, American, and Middle Eastern populations. Regarding Asia, reports abound on EB in Japanese and Chinese populations, but data for other Asian countries, including Taiwan, remain relatively scarce [3–6]. Therefore, we aimed to elucidate the molecular pathology and characterize the clinical subtypes of EB in Taiwan through a combination of approaches, including next-generation sequencing, IF studies, TEM, and other ancillary tests.

## Results

**Fig. 1:**
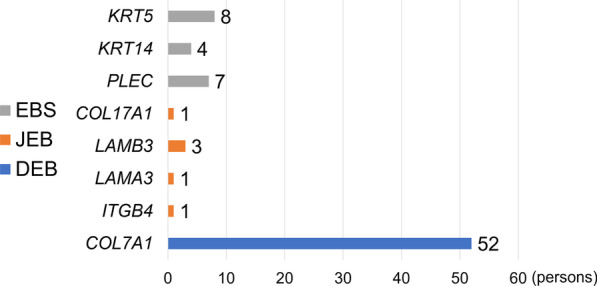
Number of EB cases by major subtypes and genes. Eight EBS patients had four mutations in *KRT5*, four EBS patients had two mutations in *KRT14*, and seven EBS patients had eleven mutations in *PLEC*. Six JEB patients had a total of 10 mutations in *LAMA3*, *LAMB3, ITGB4*, and *COL17A1*. Fifty-two DEB patients with 33 mutations in *COL7A1* were found

A total of 77 EB patients in 45 families participated in this study, including 19 patients with EBS, six patients with JEB, and 52 patients with DEB. TEM was performed in 53 patients and IF studies in 45 patients. WES was used as the first-line tool for mutational analysis in 38 families while Sanger sequencing was employed without WES for the remaining seven families. Disease-associated variants were detected by WES in 37 of the 38 families (97.4%). RNA sequencing determined the pathogenic variants in the remaining one family. Collectively, 60 mutations (22 novel and 38 recurrent) were found in *KRT5*, *KRT14*, *PLEC*, *COL17A1*, *LAMB3*, *LAMA3*, *ITGB4*, and *COL7A1* (Fig. 1 and Additional file 3: Table S1).

### Clinical subtypes and molecular pathology of EBS

Of the 19 EBS patients (12 families), eight patients had mutations in *KRT5* (42.1%), four patients had mutations in *KRT14* (21.1%), and seven patients had mutations in *PLEC* (36.8%). All patients with *KRT5*/*KRT14* mutations had autosomal dominant (AD) EBS. Two families (three patients: patient (PT) 8 in family 4, and PT11 and PT12 in family 6) had severe disease; the rest had mild to moderate disease. PT11 and PT12 (heterozygous mutation c.373C > T, p.Arg125Cys in *KRT14*) had herpetiform blistering and crusting with erythema on the trunk and extremities, as well as moderate to severe keratoderma on the soles. In addition to inflammatory blistering and palmoplantar keratoderma, PT8 (heterozygous mutation c.515 T > A, p.Ile172Asn in *KRT5*) also had growth retardation. The blistering of all three AD-EBS-severe patients improved over time.

All seven patients with mutations in *PLEC* had autosomal recessive (AR) disease, with four having clinically overt muscular dystrophy. All patients with intermediate AR-EBS with muscular dystrophy had at least one mutation in exon 31 (based on NM_000445), which encodes the rod domain of *PLEC* [7, 8]. One of the two patients of family 10 (PT17), who had compound heterozygous mutations (c.5269C > T and c.6067delG in *PLEC)*, died of respiratory infection. PT13 and PT14 with AR-EBS-intermediate (without muscular dystrophy) were unrelated and shared a common missense mutation in *PLEC*, c.956 T > C (p.Leu319Pro), whose full details were reported previously [9].

### Clinical subtypes and molecular pathology of JEB

All six patients with JEB had AR disease. Three of the six patients had mutations in *LAMB3*, while the other three had mutations in *COL17A1*, *LAMA3*, and *ITGB4*. Of the JEB patients, only PT20, affected by *LAMB3* mutations, had mild disease, presenting with blisters and erosions at trauma-prone areas. The remaining five patients had moderate to severe disease. PT25 with *LAMA3* mutations and PT24 with *ITGB4* mutations expired within a few months after birth from sepsis and respiratory failure. Both patients had aplasia cutis congenita; PT24 with *ITGB4* mutations also had pyloric atresia.

### Clinical subtypes and molecular pathology of DEB

The 52 DEB patients in our cohort consisted of 34 dominant DEB cases, 17 recessive DEB cases, and one case of severe DEB with unknown genotype. The most common subtypes were AD-DEB-pruriginosa (14/52, 26.9%), AD-DEB-localized (14/52, 26.9%), AR-DEB-severe (9/52, 17.3%), and AR-DEB-intermediate (7/52, 13.5%). Rare phenotypes of DEB included AD-DEB-self-improving (2/52, 3.8%) and AR-DEB-pruriginosa (1/52, 1.9%).

**Fig. 2:**
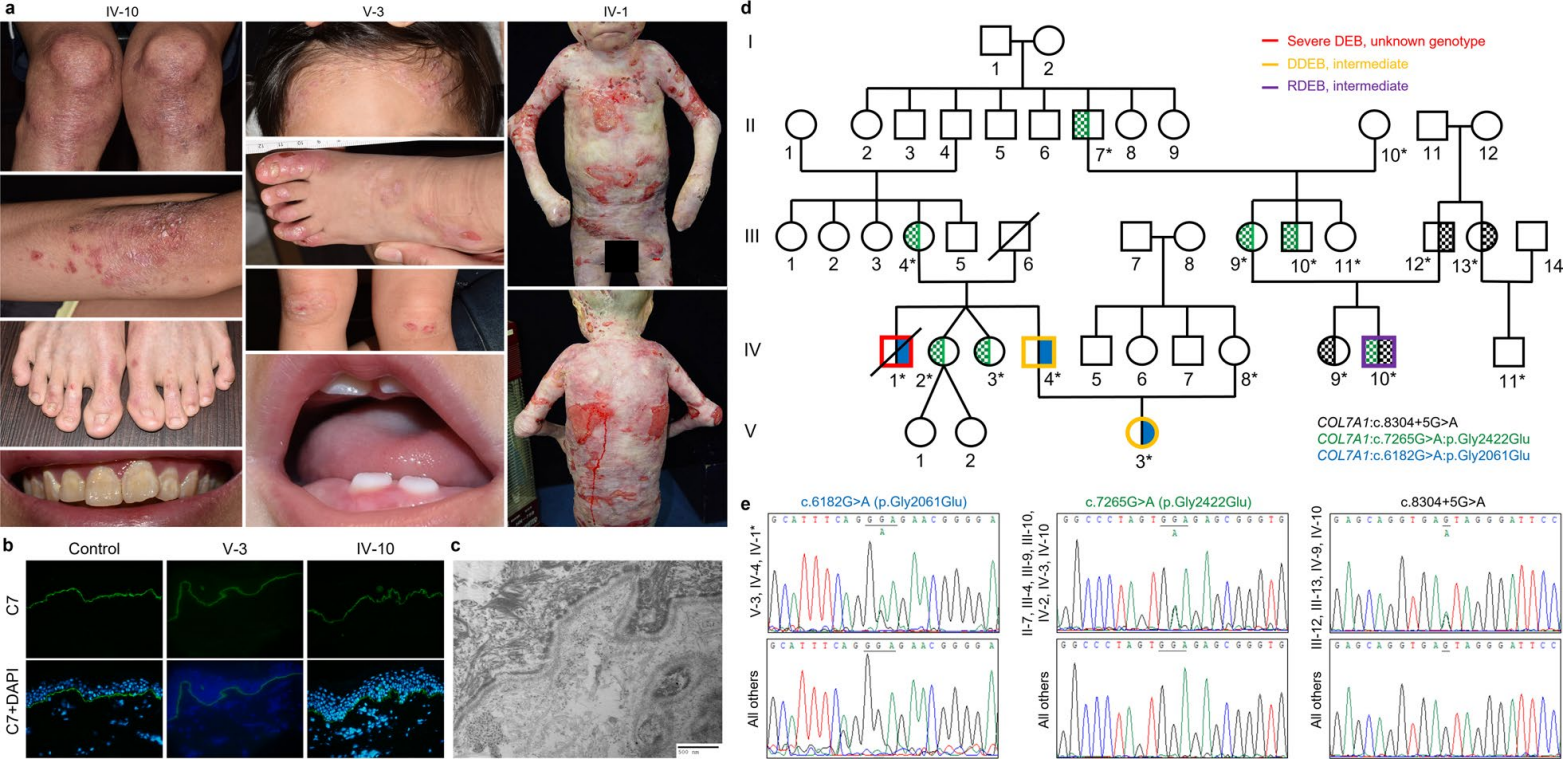
A DEB family with a possible case of dominant and recessive DEB (compound heterozygosity)-severe. **a** IV-10 (PT61) had moderate blistering, erosions, scarring, milia, nail dystrophy, and dental enamel defects. V-3 (PT60) had less severe blistering, erosions, scarring, and nail dystrophy; teeth abnormality was either absent or minimal. IV-1 (PT62) had a very severe phenotype, including pseudosyndactyly and malnutrition. **b** IF mapping showed mild reduction of C7 in both V-3 (PT60) and IV-10 (PT61) compared to their respective healthy controls (× 400 magnification). **c** TEM of V-3 (PT60) showed absent or poorly formed anchoring fibrils (40,000X magnification). The TEM findings for IV-10 (PT61) were similar. **d**, **e** V-3 (PT60) and IV-4 (PT59) shared one heterozygous mutation, c.6182G > A (p.Gly2061Glu), and both were cases of DDEB. IV-10 (PT61) was a case of RDEB with compound heterozygous mutations, c.7265G > A (p.Gly2422Glu) and c.8304 + 5G > A. (Recessive mutations are labeled as dotted half circles/squares; dominant mutations are labeled as full half circles/squares, in respective colors. Asterisks (*) indicate family members were tested for *COL7A1* mutations. The phenotype of III-6 was unknown; genomic DNA of IV-1 (PT62) was unavailable for repeat genetic testing in this study.)

There were four patients in family 31, including two AD-DEB patients (PT59 and PT60) having the heterozygous mutation, p.Gly2061Glu, one AR-DEB-intermediate patient (PT61) with compound heterozygous mutations, p.Gly2422Glu and c.8304 + 5G > A, and one case of severe DEB with unknown genotype (PT62) harboring at least a heterozygous mutation, p.Gly2061Glu (Fig. 2).

Among our DEB patients, 14 patients (seven families) had AD-DEB-pruriginosa and one patient had AR-DEB-pruriginosa. In six families, in which other subtypes of EB existed, AD-DEB-localized was the most common subtype (5/6), followed by AD-DEB-intermediate (2/6). Seven pathogenic *COL7A1* variants underlay these 15 patients, including six missense variants and one splice site variant. Patients with EB pruriginosa in this study had varying degrees of severity, ranging from extensive, confluent prurigo-like lesions on the trunk and all four extremities to prurigo lesions localized to the anterior legs only. Nail dystrophy occurred more commonly on the toenails (93 of 130 assessed nails affected) compared to the fingernails (45 of 130 assessed nails affected) (Additional file 2: Fig. S2 and Additional file 5: Table S3).

### RNA sequencing

**Fig. 3:**
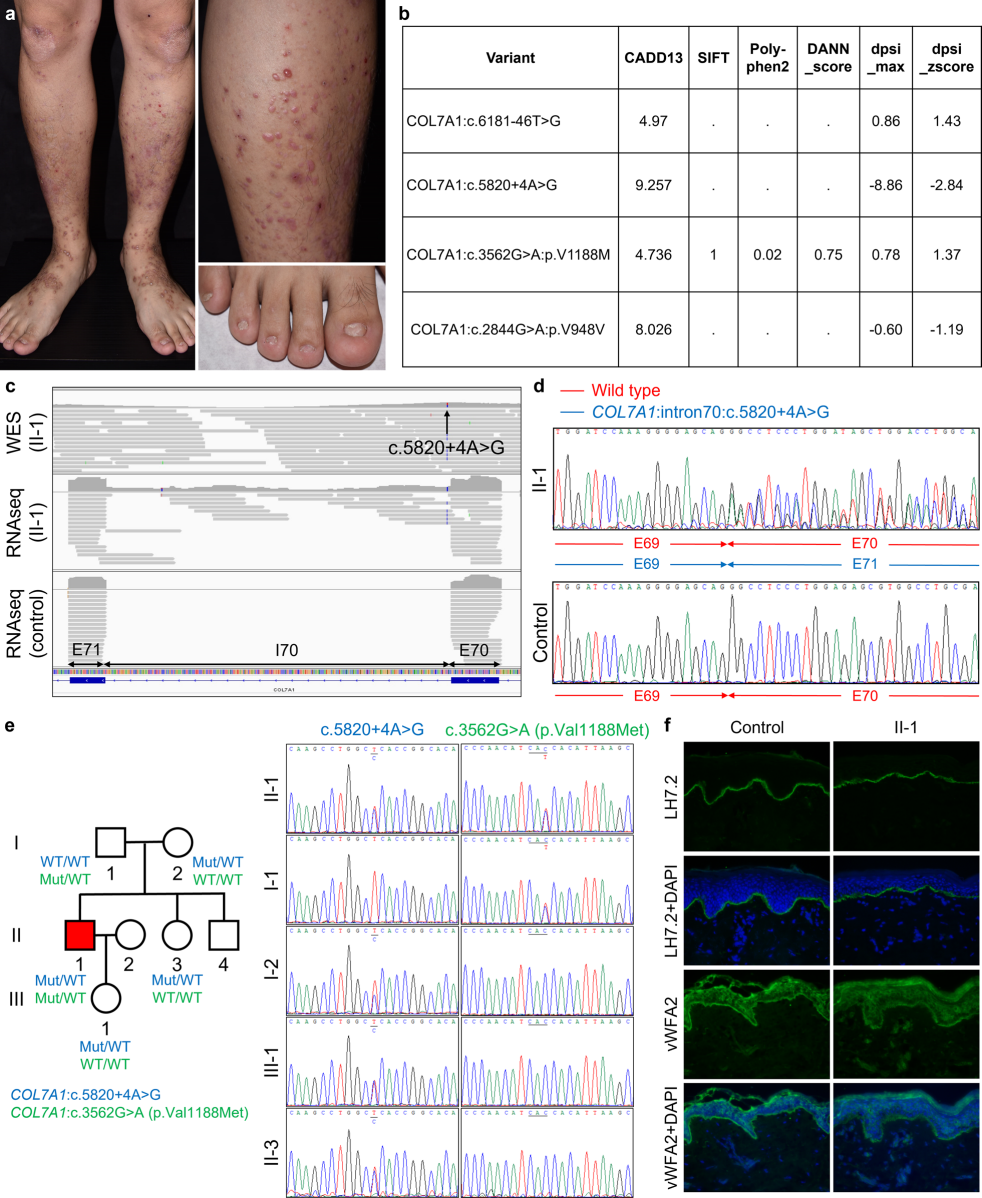
RNA sequencing, performed in addition to WES, determined pathogenic variants in PT41. **a** The proband has multiple pruritic nodules and vesicles on the lower legs as well as dystrophy of the toenails. The clinical presentation is consistent with EB pruriginosa. **b** All four disease-associated variants in *COL7A1* have CADD scores < 10. **c** RNA sequencing shows that c.5820 + 4A > G leads to the retention of intron 70 without the deletion of exon 70. **d** RT-PCR plus Sanger sequencing shows that c.5820 + 4A > G results in the deletion of exon 70 but not the retention of intron 70. **e** The proband inherited c.5820 + 4A > G from his mother and c.3562G > A from his father. **f** Staining with LH7.2, which binds to amino acid 733, is comparable between the proband and the control. In contrast, staining with a homemade polyclonal antibody that reacts with the human vWFA2 domain of C7 is reduced in the proband (× 400 magnification). This indicates that while C7 expression is not reduced, c.3562G > A (p.Val1188Met) might have caused major conformational changes in the vWFA2 region that impaired the function of C7

RNA sequencing was performed in addition to WES to confirm the pathogenicity of variants in two families. In the only AR-DEB-pruriginosa patient (PT41) in our study (Fig. 3a), WES found four potentially disease-associated variants in *COL7A1*, all with CADD (combined annotation-dependent depletion) scores < 10 (Fig. 3b). To determine the splicing effects of the candidate variants, we performed RNA sequencing, which revealed the retention of intron 70 caused by c.5820 + 4A > G (Fig. 3c) but no other aberrant splicing. In contrast, reverse transcriptase-PCR (RT-PCR) followed by Sanger sequencing showed that c.5820 + 4A > G resulted in the deletion of exon 70 without the retention of intron 70 (Fig. 3d). The discordant results of these two sequencing methods may be due to the much higher PCR efficiency of exon 70-skipped transcript over intron 70-retained transcript in the RT-PCR plus Sanger sequencing assessment.

Another *COL7A1* disease-associated variant, c.3562G > A, segregating with phenotype in the family of PT41, was presumed to lead to a valine substitution for methionine at amino acid 1188, which falls on the vWFA2 of type VII collagen (C7) (Fig. 3e). RT-PCR and Sanger sequencing of cDNA found no aberrant splicing. To further investigate the pathogenicity of c.3562G > A (p.Val1188Met), we used a polyclonal antibody for murine vWFA2 previously used by Iwata et al., which also reacted with the human vWFA2 domain of C7, for IF studies [10]. While C7 staining was comparable between healthy controls and PT41 using LH7.2 antibody, C7 staining was reduced with the antibody targeting vWFA2. This indicates that while overall C7 expression based on LH7.2 expression was not reduced, c.3562G > A (p.Val1188Met) might have caused conformational changes in the vWFA2 region that were detrimental to the function of C7 (Fig. 3f).

**Fig. 4:**
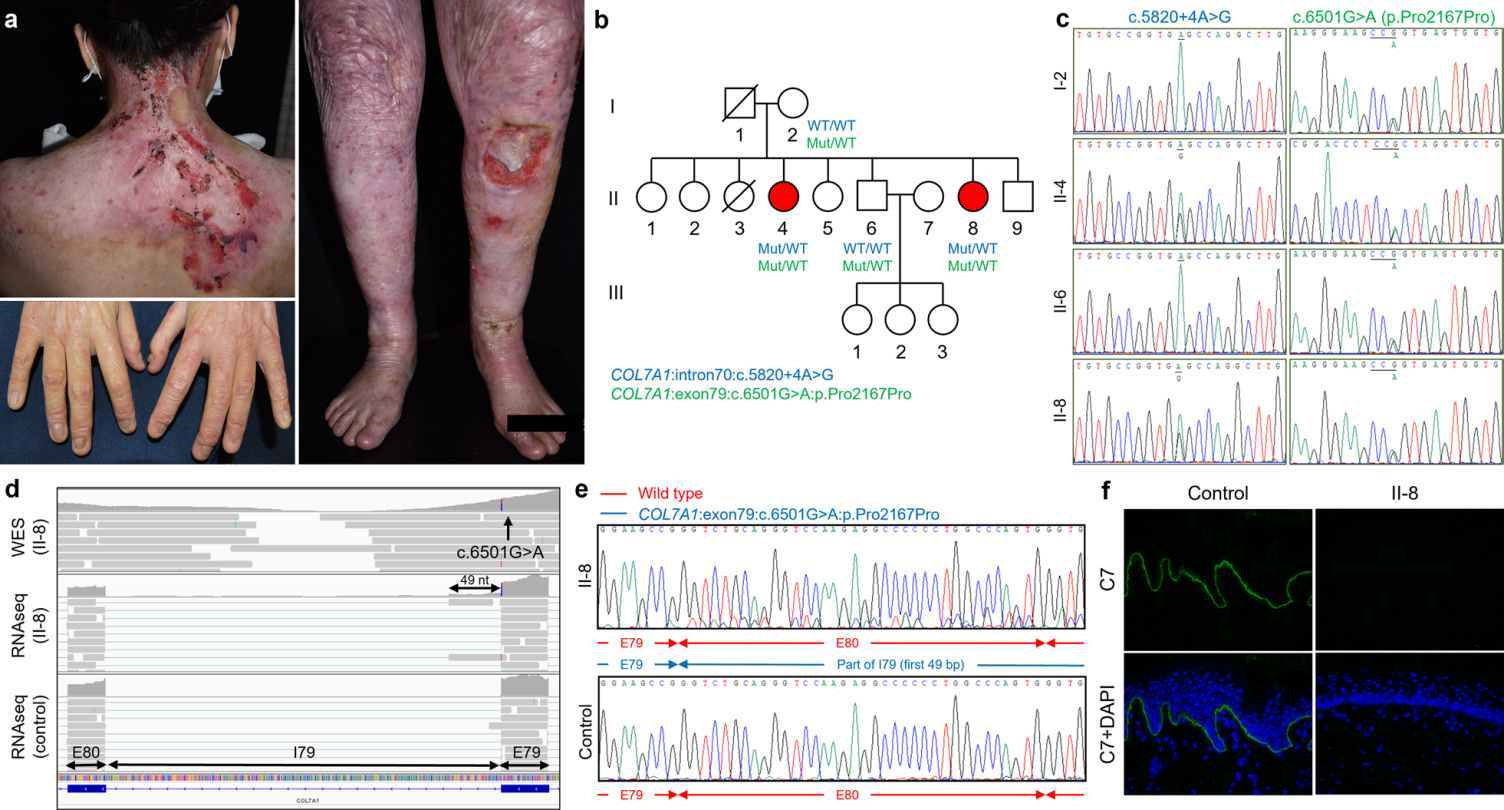
RNA sequencing, performed in addition to WES, showed results consistent with RT-PCR plus Sanger sequencing in PT70. **a** The proband (PT70/II-8) has extensive erosions, chronic wounds, scarring of the skin, and nail dystrophy. The clinical presentation is consistent with severe DEB. **b**, **c** Both II-4 and II-8 have the compound heterozygous mutations, c.5820 + 4A > G and c.6501G > A in *COL7A1*. **d**, **e** RNA sequencing shows that c.6501G > A leads to the retention of part of intron 79 (49 nts); RT-PCR plus Sanger sequencing reveals similar findings. **f** Staining of C7 is markedly reduced compared to the control (× 400 magnification)

RNA sequencing, as well as RT-PCR plus Sanger sequencing, was also performed in mutational analysis of PT70, who had AR-DEB-severe (Fig. 4a). In PT70, WES identified two disease-associated variants in *COL7A1*: c.6501G > A and c.5820 + 4A > G (Fig. 4b, c). Both variants were located at exon–intron junctions and were predicted to lead to aberrant splicing (z-score of the dPSI relative (dpsi_zscores): -2.664 and -2.846, respectively). Both RNA sequencing and RT-PCR plus Sanger sequencing showed that c.6501G > A led to the retention of the first 49 nucleotides (nts) of intron 79 (Fig. 4d, e). In contrast, the two sequencing methods showed discordant results for c.5820 + 4A > G in PT70, as in PT41.

### Splice-site mutations and their consequences

We found a total of 13 splice-site mutations, including five novel mutations and eight reported mutations (Additional file 3: Table S1). The influence on splicing of six of the 13 splice-site mutations was unknown. To investigate the consequences of these splice-site mutations, we performed RNA extraction from the patient’s skin, followed by RT-PCR and Sanger sequencing and/or RNA sequencing. The results are summarized in Additional file 4: Table S2. Four of the 13 splice-site mutations led to skipping of certain exon(s) without disrupting the reading frame; seven caused a frameshift and the formation of premature termination codons (PTCs); two resulted in multiple alternative splice forms. The results of our mRNA analyses on seven of the 13 mutations were consistent with previous work.

## Discussion

In this study, we systematically investigated the clinical subtypes and molecular pathologies of 77 Taiwanese EB patients through WES, Sanger sequencing, TEM, and IF studies. As the initial tool for mutational analysis, WES correctly identified disease-associated variants in 37 of the 38 families (97.4%).

In recent years, next-generation sequencing (NGS) has become a first-line tool for mutational analysis for many genodermatoses, including EB. Such an approach has been proven feasible by several studies utilizing NGS with EB-specific multigene panels [11–15], yielding a diagnostic rate of 83.5–97.7%, depending on the study population and the gene panel used. Our results are consistent with such previous studies.

Of the 38 families in which WES was used as a first-line tool for mutational analysis, one family (the family of PT41) required the additional use of RNA sequencing to determine the pathogenic mutations. In this case, we used RNA sequencing to determine whether each disease-associated variant (all with CADD scores < 10) found by WES affected splicing. The results helped establish the diagnosis of AR-EB-pruriginosa, despite atypical IF results with conventional LH7.2 antibody for C7. Similar approaches utilizing RNA sequencing to study the transcriptomic changes of specific variants found by WES had also been used in mutational analysis for EB [16, 17]. Vahidnezhad et al. also used RNA sequencing to prove coexistence of both recessive simplex and junctional EB phenotypes in one patient with homozygous mutations in both *EXPH5* and *COL17A1* [16].

Interestingly, the results of transcriptomic analysis of c.5820 + 4A > G in PT41 with RNA sequencing and RT-PCR plus Sanger sequencing were discordant, with RNA sequencing showing intron 70-retained transcripts and PCR plus Sanger sequencing showing exon 70-skipped transcripts caused by c.5820 + 4A > G. Similar results were also seen in the mutational analysis of PT70, who shared this mutation. We believe that the higher amplification efficiency of exon 70-skipped transcripts due to the primers used in RT-PCR might have resulted in this disparity, and hence, the "biased" results of RT-PCR plus Sanger sequencing. The advantage of sequencing splice transcripts without preference, along with the ability to uncover deep intronic, silent, and synonymous exonic variants often overlooked by WES [18], makes RNA sequencing a useful additional technique to WES in the mutational analysis of genodermatoses.

Our study included 19 EBS patients (24.7%), six JEB patients (7.8%), and 52 DEB patients (67.5%). In contrast, most studies on the prevalence of EB using national EB registries showed EBS to be the most common subtype, accounting for over or close to 50% of all EB cases [19–21]. Published studies also revealed that EBS as a share of EB is highest in Northern Ireland (88%), followed by Scotland (58%), Australia (56%), the United States (54%), Japan (51%), and Norway (43%), with the lowest occurring in Croatia (16%) [20, 22]. Considering this, EBS seems under-represented and DEB over-represented in the current study. Such deviation from the world's EB epidemiology data could be explained by the fact that our study, which utilized data of patients who came to a tertiary hospital, probably selected for a more severe EB population. EBS, in which a large percentage being mild or self-improving, have a less severe phenotype in general, making patients with EBS less likely to seek medical attention and genetic counseling than DEB patients. Several other studies conducted in similar hospital settings also showed underrepresentation of EBS and overrepresentation of DEB [12, 13, 23].

In our study, only PT25 (JEB) and PT77 (AR-DEB-severe) had disease caused by homozygous mutations. This is expected because the rate of consanguineous marriages is low in Taiwan. In countries where consanguineous marriages are much more common, such as Iran and Kuwait, EB is usually inherited in a recessive mode and mutations are more frequently found at homozygous status. In addition, EB is caused by mutations in genes that usually are more rarely mutated in the disease [14, 24]. Indeed, in addition to the low prevalence of homozygous mutations in Taiwan, our study did not identify recurrent mutations suggestive of common ancestral alleles in our population study, either.

The phenotypes of the 12 patients with EBS caused by *KRT5* or *KRT14* mutations in our study correlated well with their genotypes. Patients with mutations lying in the highly conserved boundary motif of keratin 5 and keratin 14 demonstrated severe phenotypes; patients with mutations elsewhere had much less severe blistering. All three patients with AD-EBS-severe improved over time, presenting less acute lesions (blisters and erosions) and more hyperpigmentation.

The 52 DEB patients in our study included 34 patients with dominant DEB, 17 patients with recessive DEB, and one patient with severe DEB but unknown genotype. These patients had a total of 33 mutations, including 12 novel mutations. Nine mutations were found in unrelated Taiwanese families; the most frequent ones were p.Gly2043Arg and p.Pro1805Leu, both occurring in three families, the former of which being the most common glycine substitution mutation underlying dominant DEB worldwide [25, 26], leading to both reduced secretion of pro-C7 into the extracellular matrix and increased enzymatic susceptibility of C7 [26].

c.5414C > T (p.Pro1805Leu) in *COL7A1* was a novel mutation seemingly specific to Taiwanese EB populations. The substitution of leucine in this mutation occurred on the Y residue of a Gly-X–Y repeat in exon 62. Since the proline at this residue is often hydroxylated to 4-hydroxyproline and involved in stabilization of collagen triple helices, this mutation might disrupt the thermal stability of the triple helices [27, 28]. Interestingly, within the three EB families with this mutation, all heterozygous carriers had a normal phenotype, suggesting a recessive nature of this mutation.

In general, the genotypes in our DEB cohort correlated relatively well with established genotype–phenotype correlations. In most of the AR-DEB-severe patients, the disease was caused by biallelic nonsense, frameshift, and certain splice-site mutations, all resulting in PTCs [29]. However, in two AR-DEB-severe patients (PT70, PT71), the disease was caused by one splice-site mutation causing PTC and the other causing inframe exon skipping. Still, none of the AR-DEB-intermediate patients in our study had biallelic nonsense mutations.

In the patient with severe DEB but unknown genotype (PT62), only one mutation, c.6182G > A (p.Gly2061Glu), was found by Sanger sequencing. Further analysis was not possible because the patient had died of the disease. This patient had an extremely severe phenotype, characterized by extensive blistering, scarring, growth retardation, flexure contractures, and pseudosyndactyly. In addition to p.Gly2061Glu, the patient might have had another recessive glycine substitution mutation, p.Gly2422Glu, based on its presence in the proband’s mother and siblings. Although uncommon, AR-DEB-severe could be caused by missense mutations only; the homozygosity of both c.7705G > C (p.Gly2569Arg) and c.8245G > A (p.Gly2749Arg) caused a severe phenotype in two families [30]. Nevertheless, due to a lack of direct mutational data, it was unknown whether the severe phenotype of PT62 resulted from the compound heterozygosity of the two missense mutations, an unidentified mutation in *COL7A1*, or other disease modifiers.

Fifteen of the 52 DEB patients (15/52, 28.8%) had EB pruriginosa in this study, including 14 AD-DEB-pruriginosa patients and one AR-DEB-pruriginosa patient. Typically, EB pruriginosa presents with intensely pruritic excoriated nodules, papules, and plaques on the extensor aspects of the extremities, while more generalized lesions are seen in some patients. The disease can be dominant or recessive, but the dominant form is more common [31]. EB pruriginosa is traditionally considered a rare subtype of EB, and the largest series of EB pruriginosa reported to date consisted of eight patients without mutational data [32], while some cases were reported under other names, including pretibial EB [5], a term used in older classifications. The large number of EB-pruriginosa patients reported by Lee et al. from Taiwan [5] and by our group indicates that EB pruriginosa is a relatively common subtype of DEB in Taiwan.

In our study, all 14 patients with AD-DEB-pruriginosa had glycine substitution mutations. This is consistent with a systemic review by Kim et al., which found glycine substitution mutations (52.7%) and in-frame skipping (33.8%) to be the most common mutations underlying EB pruriginosa [33]. It is noteworthy that mutations associated with EB pruriginosa in our studies showed marked inter-familial and intra-familial variations in phenotype. Of the six families in which other subtypes of DEB occurred, the same pathogenic variants resulted in AD-DEB-localized in five (5/6) and AD-DEB-intermediate in two families (2/6). Modifiers that led to a phenotypic difference of the same mutation could be genetic, epigenetic, or environmental, and remain mostly unknown [2].

## Conclusions

This study is the first large-scale attempt at clinical subtyping and mutational analysis of Taiwanese EB patients. It confirmed that WES has a high diagnostic rate as the first-line tool for mutational analysis of EB and showed that RNA sequencing was complementary to WES in cases with multiple potential splice-site mutations. In addition to expanding the spectrum of EB mutations, we also investigated the consequences of 11 splice-site mutations. Such clinical and molecular data provides a foundation for clinical decisions and the development of new therapies.

## Methods

### Collection of clinical information and patient subtyping

This study was approved by the Institutional Review Board of the National Cheng Kung University Hospital (IRB number: A-BR-104-052) and was carried out in accordance with the Declaration of Helsinki and local ethics requirements. A schematic outline summarizes the research methods (Additional file 1: Figure S1). National Cheng Kung University Hospital is a national referral center for EB in Taiwan. Data on cutaneous and extracutaneous manifestations of individual EB patients and their families from January 1, 2012, to June 1, 2021, were collected through direct interviews and a review of medical records. Subtyping of individual patients was done based on clinical, molecular, and genetic grounds using the “onion skin” approach, according to the 2020 EB consensus reclassification [2].

### Pathologic examinations

For patients who had not had diagnostic tests prior to referral, we performed skin biopsies for routine histopathology, TEM, and IF studies for EB-specific proteins. Usually, a shave biopsy was performed on intact skin after the area was stroked 20–30 times with an index finger to elicit a fresh blister or cleavage plane. This shave biopsy technique allowed us to examine vital structures in the skin down to the level of the superficial dermis, which was sufficient for diagnosing EB.

### Mutational analysis

If the genetic test results were not already available, mutational analysis was performed using DNA extracted from 2 to 4 ml of peripheral blood from the patient and related family members, with informed consent. We used WES as a first-line tool and focused on the 16 genes implicated in classical forms of EB [2], plus *DSP*, *PKP1*, *JUP*, and *TGM5* to screen for disease-associated variants. These additional four genes were listed as EB genes in the international consensus classification of EB established in 2014 [2, 34]. However, mutations in these additional genes were reclassified as causing “other EB-related disorders with skin fragility” in 2020 [2]. Exome libraries were generated with SureSelect Human All Exon V6 (Agilent, Santa Clara, CA, U.S.A.) and sequenced with 2 × 100 paired-end sequencing on the NextSeq500 platform (Illumina, San Diego, CA, U.S.A.). We performed variant calling by using a previously published in‐house pipeline [35], and cross-referenced the identified variants with publicly available variant data (ExAC, gnomAD, and the 1000 genome project). Variants with a frequency of less than 0.05% were considered potentially pathogenic. These variants were then confirmed using Sanger sequencing, followed by segregation analysis in DNA of related family members. Potentially pathogenic variants that were able to explain the patient’s clinical manifestations and laboratory test results were considered as likely to account for the disease.

### Investigation on the pathogenicity of splice-site and missense variants

In some patients, potential pathogenic variants were novel missense variants and splice-site mutations. Since the pathogenicity of these mutations was not straightforward, we performed RT-PCR plus Sanger sequencing, and/or RNA sequencing, to study the effect of splice-site variants. RNA sequencing was performed on the Illumina NovaSeq 6000 platform (Illumina, USA). We used fastp to trim and filter raw sequencing reads [36]. Processed reads were aligned to the hg37 human reference genome in HISAT2 [37]. We analyzed the resulting BAM files by the featureCounts function of the Subread tool [38] such that alignments with a score of 10 or less were removed, and only reads with unique mappings were counted. The counts matrix was annotated using the hg37 NCBI RefSeq file. For one missense variant, p.Val1188Met in *COL7A1*, we performed IF studies with a non-commercial polyclonal antibody targeting the vWFA2 domain of human C7 to study its pathogenicity [10].

## Supplementary Information

**Additional file 1. Supplementary Figure 1.** Schematic outline of research methods.

**Additional file 2. Supplementary Figure 2**. Varying degrees of severity of EB pruriginosa, from relatively mild to severe.

**Additional file 3. Supplementary Table 1.** Mutations and clinical subtypes of EB in Taiwan.

**Additional file 4. Supplementary Table 2.** Consequences of splice site mutations.

**Additional file 5. Supplementary Table 3.** Genotype and clinical presentation of patients with EB pruriginosa.

## Data Availability

The data that support the findings of this study are available from the corresponding author upon reasonable request.
