# Supplementary material for: Mutational analysis of epidermolysis bullosa in Taiwan by whole-exome sequencing complemented by RNA sequencing: a series of 77 patients
Source: Orphanet J Rare Dis. 2022 Dec 28;17:451. doi: 10.1186/s13023-022-02605-1 (PMC9795651; doi:10.1186/s13023-022-02605-1)
Supplement: Supplementary file 1 — Additional file 1. Supplementary Figure 1. Schematic outline of research methods. [file 13023_2022_2605_MOESM1_ESM.docx]

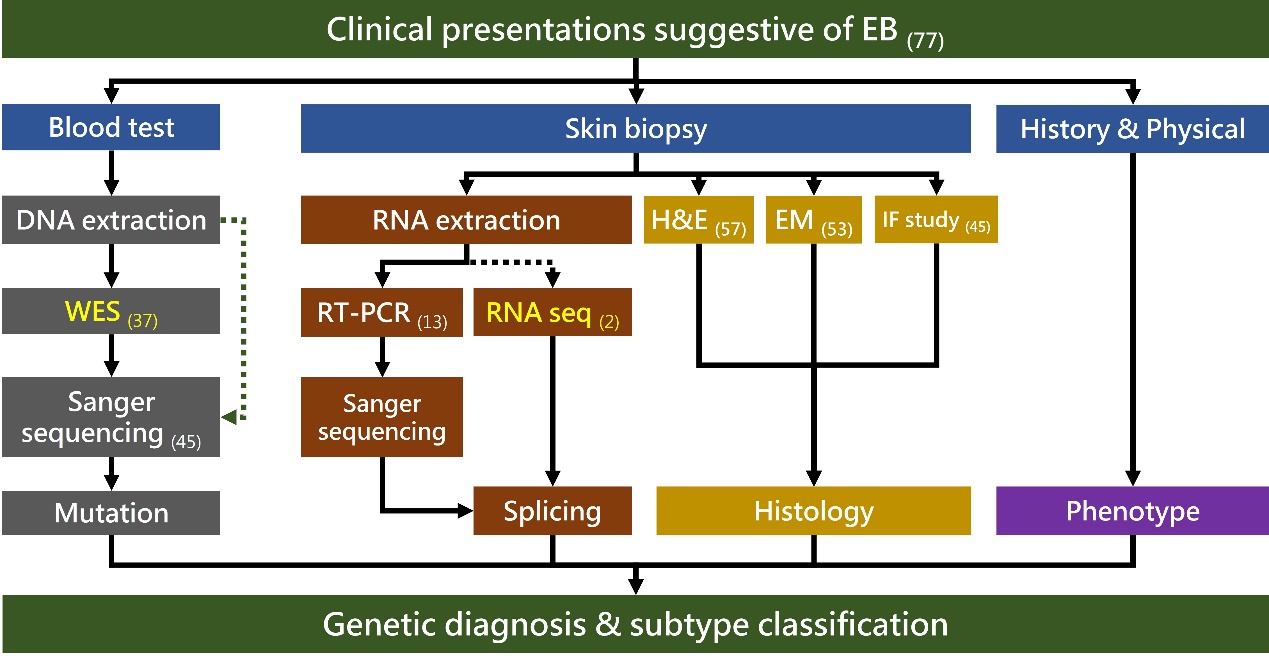


**Supplementary Figure 1. Schematic outline of research methods.** Mutational analysis, if not previously performed, was done on all EB patients using whole exome sequencing (WES) as a first-line tool for diagnosis. The results were confirmed by Sanger sequencing and segregation analysis. Skin biopsies were performed to harvest samples for routine histopathology, transmission electron microscopy (TEM), immunofluorescence (IF) studies, and RNA analysis. Patient phenotypes were recorded, including the mode of inheritance, the severity of cutaneous manifestations, and the extracutaneous manifestations. (Dotted arrows indicate alternative methods for analysis; lowercase numbers in brackets indicate the number of families receiving corresponding tests. RT-PCR: reverse transcriptase-PCR, RNA seq: RNA sequencing, H&E: hematoxylin and eosin)
