# Supplementary material for: Mutational analysis of epidermolysis bullosa in Taiwan by whole-exome sequencing complemented by RNA sequencing: a series of 77 patients
Source: Orphanet J Rare Dis. 2022 Dec 28;17:451. doi: 10.1186/s13023-022-02605-1 (PMC9795651; doi:10.1186/s13023-022-02605-1)
Supplement: Supplementary file 2 — Additional file 2. Supplementary Figure 2. Varying degrees of severity of EB pruriginosa, from relatively mild to severe. [file 13023_2022_2605_MOESM2_ESM.docx]

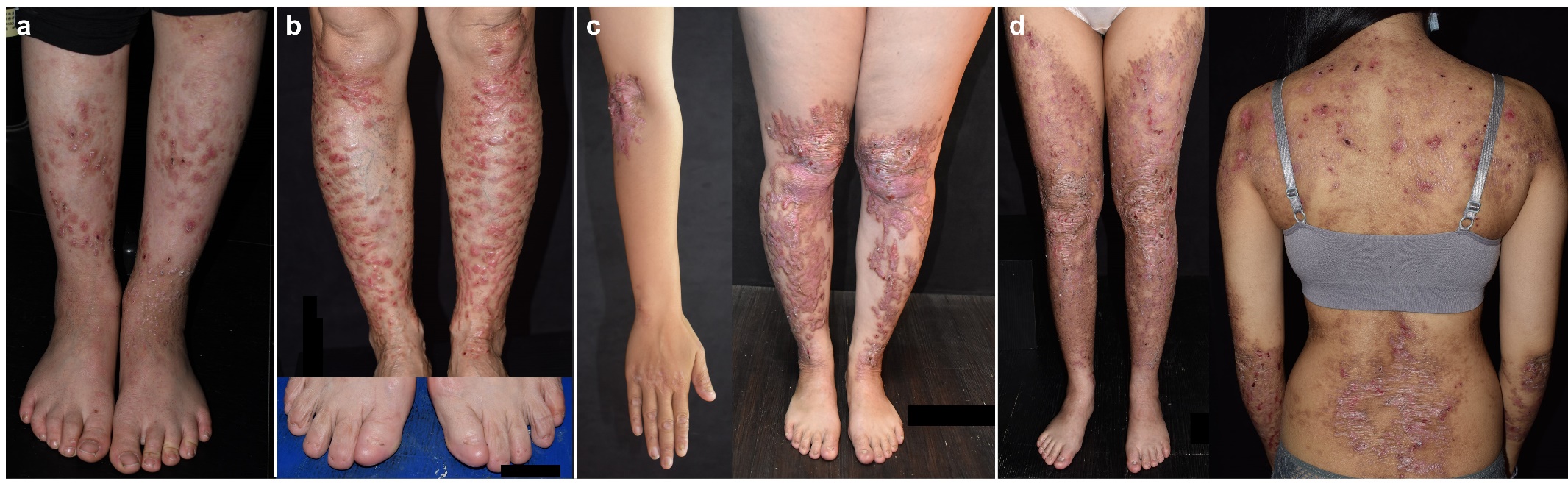


**Supplementary Figure 2. Varying degrees of severity of EB pruriginosa, from relatively mild to severe**

EB pruriginosa is characterized by intensely pruritic excoriated nodules, papules, and plaques on the extensor aspects of the extremities. (a) PT47 has erythematous to violaceous nodules and papules, sometimes confluent, which are localized to the lower legs. No nail dystrophy is present. (b) Lesions of PT37 are still localized to the lower legs, but they are more prominent, forming linear plaques. All toenails were dystrophic. (c) PT34 has thicker and more excoriated lesions, with occurrence of lesions on other trauma prone sites, such as the elbows. Nail dystrophy is also present. (d) PT40 has extensive excoriated nodules and plaques involving the legs, the upper extremities, and the trunk. Nails are also dystrophic.
