# Supplementary material for: Mutational analysis of epidermolysis bullosa in Taiwan by whole-exome sequencing complemented by RNA sequencing: a series of 77 patients
Source: Orphanet J Rare Dis. 2022 Dec 28;17:451. doi: 10.1186/s13023-022-02605-1 (PMC9795651; doi:10.1186/s13023-022-02605-1)
Supplement: Supplementary file 3 — Additional file 3. Supplementary Table 1. Mutations and clinical subtypes of EB in Taiwan. [file 13023_2022_2605_MOESM3_ESM.docx]

**Supplementary table 1. Mutations and clinical subtypes of EB in Taiwan**

| Family | Patient | EB subtype | Gene | Mutation | Zygosity | ACMG scoring | Reference |
| --- | --- | --- | --- | --- | --- | --- | --- |
| 1 | 1  2  3 | AD-EBS, localized | *KRT5* | c.119G>T (p.Gly40Val) | Het | Likely pathogenic | Novel |
| 2 | 4  5  6 | AD-EBS, intermediate | *KRT5* | c.504G>C (p.Glu168Asp) | Het | Pathogenic | [1] |
| 3 | 7 | AD-EBS, intermediate | *KRT5* | c.1401C>G (p.Ile467Met) | Het | Pathogenic | [2] |
| 4 | 8 | AD-EBS, severe | *KRT5* | c.515T>A (p.Ile172Asn) | Het | Pathogenic | [3] |
| 5† | 9  10 | AD-EBS, localized | *KRT14* | c.1231_1233delGAG (p.Glu411del) | Het | Pathogenic | [4] |
| 6† | 11  12 | AD-EBS, severe | *KRT14* | c.373C>T (p.Arg125Cys) | Het | Pathogenic | [5] |
| 7 | 13 | AR-EBS, intermediate | *PLEC* | c.6955C>T (p.Arg2319Ter) c.956T>C (p.Leu319Pro) | Het Het | Pathogenic  Uncertain significance | [6]  [7] |
| 8 | 14 | AR-EBS, intermediate | *PLEC* | c.2807G>A (p.Trp936Ter) c.956T>C (p.Leu319Pro) | Het Het | Likely pathogenic  Uncertain significance | [7] [7] |
| 9 | 15 | AR-EBS, intermediate | *PLEC* | c.7699C>T (p.Gln2567Ter) c.12566T>C (p.Leu4189Pro) | Het Het | Likely pathogenic  Uncertain significance | Novel Novel |
| 10† | 16  17 | AR-EBS, intermediate with muscular dystrophy | *PLEC* | c.5269C>T (p.Gln1757Ter) c.6067delG (p.Ala2023ProfsTer19) | Het Het | Likely pathogenic  Likely pathogenic | Novel Novel |
| 11 | 18 | AR-EBS, intermediate with muscular dystrophy | *PLEC* | c.7390C>T (p.Gln2464Ter) c.4294_4306del (p.Glu1432fs) | Het Het | Pathogenic  Likely pathogenic | Novel Novel |
| 12† | 19 | AR-EBS, intermediate with muscular dystrophy | *PLEC* | c.6991C>T (p.Gln2331Ter) c.194-1G>C | Het Het | Pathogenic  Likely pathogenic | Novel Novel |
| 13 | 20 | JEB, localized | *LAMB3* | c.2137+1G>T c.629-12T>A | Het Het | Pathogenic  Likely pathogenic | [8]  [3] |
| 14 | 21 | JEB, intermediate | *LAMB3* | c.3119G>A (p.Trp1040Ter) c.373-9T>A | Het Het | Pathogenic  Uncertain significance | [3] [9] |
| 15 | 22 | JEB, intermediate | *LAMB3* | c.3512G>A (p.Cys1171Tyr)  c.629-12T>A | Het  Het | Uncertain significance  Likely pathogenic | [3]  [3] |
| 16 | 23 | JEB, intermediate | *COL17A1* | c.2566C>T (p.Gln856Ter) c.2003-1G>C | Het Het | Likely pathogenic  Likely pathogenic | [10] [11] |
| 17 | 24 | JEB, with pyloric atresia | *ITGB4* | c.121T>C (p.Cys41Arg) c.3719G>A (p.Trp1240Ter) | Het Het | Likely pathogenic  Pathogenic | Novel [12] |
| 18 | 25 | JEB, severe | *LAMA3* | c.2991_2992del (p.Gly998LeufsTer25) | Hom | Likely pathogenic | [13] |
| 19 | 26 | AD-DEB, self-improving | *COL7A1* | c.4439G>A (p.Gly1480Asp) | Het | Pathogenic | [3] |
| 20 | 27 | AD-DEB, self-improving | *COL7A1* | c.6074G>A (p.Gly2025Asp) | Het | Pathogenic | [14] |
|  | 28 | AD-DEB, localized |  |  |  |  |  |
| 21 | 29  30  31 | AD-DEB, localized | COL7A1 | c.6127G>A (p.Gly2043Arg) | Het | Pathogenic | [15] |
| 22 | 32 | AD-DEB, localized | *COL7A1* | c.5327G>A (p.Gly1776Glu) | Het | Likely pathogenic | [16] |
| 23 | 33 | AD-DEB, localized | COL7A1 | c.6127G>A (p.Gly2043Arg) | Het | Pathogenic | [15] |
|  | 34 | AD-DEB, pruriginosa |  |  |  |  |  |
| 24 | 35  36 | AD-DEB, localized | *COL7A1* | c.7697G>A (p.Gly2566Glu) | Het | Likely pathogenic | Novel |
|  | 37 | AD-DEB, pruriginosa |  |  |  |  |  |
| 25 | 38  39 | AD-DEB, localized | *COL7A1* | c.6182G>A (p.Gly2061Glu) | Het | Pathogenic | [17] |
|  | 40 | AD-DEB, pruriginosa |  |  |  |  |  |
| 26 | 41 | AR-DEB, pruriginosa | *COL7A1* | c.5820+4A>G  c.3562G>A (p.Val1188Met) | Het  Het | Likely pathogenic  Uncertain significance | Novel  Novel |
| 27 | 42 | AD-DEB, localized | *COL7A1* | c.4670G>A (p.Gly1557Glu) | Het | Pathogenic | [18] |
|  | 43  44  45  46  47  48 | AD-DEB, pruriginosa |  |  |  |  |  |
| 28 | 49  50 | AD-DEB, pruriginosa | *COL7A1* | c.5318G>T (p.Gly1773Val) | Het | Pathogenic | [19] |
| 29 | 51 | AD-DEB, pruriginosa | *COL7A1* | c.6127G>A (p.Gly2043Arg) | Het | Pathogenic | [15] |
|  | 52 | AD-DEB, intermediate |  |  |  |  |  |
| 30 | 53 | AD-DEB, localized | *COL7A1* | c.5318G>T (p.Gly1773Val) | Het | Pathogenic | [19] |
|  | 54 |  |  |  |  |  |  |
|  | 55 |  |  |  |  |  |  |
|  | 56 | AD-DEB, intermediate |  |  |  |  |  |
|  | 57 | AD-DEB, pruriginosa |  |  |  |  |  |
|  | 58 |  |  |  |  |  |  |
| 31 | 59  60 | AD-DEB, intermediate | *COL7A1* | c.6182G>A (p.Gly2061Glu) | Het | Pathogenic | [17] |
|  | 61 | AR-DEB, intermediate |  | c.7265G>A (p.Gly2422Glu) c.8304+5G>A | Het Het | Likely pathogenic  Pathogenic | [3] Novel |
|  | 62* | Severe DEB, genotype unknown |  | c.6182G>A (p.Gly2061Glu) Unknown | Het Unknown | Pathogenic  Unknown | [17] Unknown |
| 32 | 63 | AR-DEB, intermediate | *COL7A1* | c.6394-1G>A c.6022C>T (p.Arg2008Cys) | Het Het | Likely pathogenic  Pathogenic | Novel [20] |
| 33 | 64 | AR-DEB, intermediate | *COL7A1* | c.7234C>T (p.Arg2412Ter) c.5499C>T | Het Het | Pathogenic  Uncertain significance | [21] [22] |
| 34 | 65 | AR-DEB, intermediate | *COL7A1* | c.5414C>T (p.Pro1805Leu) c.7769delG (p.Gly2590ValfsTer41) | Het Het | Likely pathogenic  Likely pathogenic | Novel [17] |
| 35 | 66 | AR-DEB, intermediate | *COL7A1* | c.5414C>T (p.Pro1805Leu)  c.1732C>T (p.(Arg578Ter) | Het Het | Likely pathogenic  Pathogenic | Novel [23] |
| 36 | 67 | AR-DEB, intermediate | *COL7A1* | c.5414C>T (p.Pro1805Leu) c.4719delA (p.Glu1573AspfsTer137) | Het Het | Likely pathogenic  Likely pathogenic | Novel Novel |
| 37 | 68 | AR-DEB, intermediate | *COL7A1* | c.7272G>A c.7265G>A (p.Gly2422Glu) | Het Het | Likely pathogenic  Likely pathogenic | Novel [3] |
| 38 | 69 | AR-DEB, severe | *COL7A1* | c.5532+5G>A c.4719delA (p.Glu1573AspfsTer137) | Het Het | Likely pathogenic  Likely pathogenic | [24] Novel |
| 39 | 70  71 | AR-DEB, severe | *COL7A1* | c.6501G>A c.5820+4A>G | Het Het | Pathogenic  Likely pathogenic | [25] Novel |
| 40 | 72 | AR-DEB, severe | *COL7A1* | c.5532+5G>A c.2091delC (p.Ser698AlafsTer56) | Het Het | Likely pathogenic  Likely pathogenic | [24] Novel |
| 41† | 73 | AR-DEB, severe | *COL7A1* | c.3372_3373insGG (p.Pro1125GlyfsTer37)  c.7769delG (p.Gly2590ValfsTer41) | Het Het | Likely pathogenic  Likely pathogenic | [17] [17] |
| 42 | 74 | AR-DEB, severe | *COL7A1* | c.8569G>T (p.Glu2857Ter) c.7289delC (p.Pro2430GlnfsTer36) | Het Het | Pathogenic  Pathogenic | [26] [22] |
| 43† | 75 | AR-DEB, severe | *COL7A1* | c.4603G>T (p.Glu1535Ter) c.8407+5G>C | Het Het | Pathogenic  Pathogenic | [17] [17] |
| 44 | 76 | AR-DEB, severe | *COL7A1* | c.7488delG (p.Pro2498LeufsTer18) c.7486G>A (p.Gly2496Arg) c.5107G>T (p.Gly1703Ter) | Het Het Het | Likely pathogenic  Pathogenic  Pathogenic | Novel Novel Novel |
| 45† | 77 | AR-DEB, severe | *COL7A1* | c.1573C>T (p.Arg525Ter) | Hom | Pathogenic | [27] |

*Besides c.6182G>A (p.Gly2061Glu), this patient was presumed to have another mutation, c.7265G>A (p.Gly2422Glu), leading to the severe phenotype (dominant and recessive DEB). However, further genetic testing to confirm this was not possible because the patient had passed away.

Reference sequences used for annotation of mutations/variants in individual genes are as follows: NM_000424 for *KRT5*, NM_000526 for *KRT14*, NM_000445 for *PLEC*, NM_000227 for LAMA3, NM_000228 for *LAMB3*, NM_000494 for *COL17A1*, NM_000213 for *ITGB4*, NM_000094 for *COL7A1*.

†In these cases, the first-line tool for mutational analysis was Sanger sequencing rather than WES.

***References***

1. Shinkuma S, Nishie W, Jacyk WK, Natsuga K, Ujiie H, Nakamura H, et al. A novel keratin 5 mutation in an African family with epidermolysis bullosa simplex indicates the importance of the amino acid located at the boundary site between the H1 and coil 1A domains. Acta Derm Venereol. 2013;93(5):585-7.

2. Pfendner EG, Sadowski SG, Uitto J. Epidermolysis bullosa simplex: recurrent and de novo mutations in the KRT5 and KRT14 genes, phenotype/genotype correlations, and implications for genetic counseling and prenatal diagnosis. J Invest Dermatol. 2005;125(2):239-43.

3. Chen F, Huang L, Li C, Zhang J, Yang W, Zhang B, et al. Next-generation sequencing through multigene panel testing for the diagnosis of hereditary epidermolysis bullosa in Chinese population. Clin Genet. 2020;98(2):179-84.

4. Müller FB, Küster W, Wodecki K, Almeida H, Jr., Bruckner-Tuderman L, Krieg T, et al. Novel and recurrent mutations in keratin KRT5 and KRT14 genes in epidermolysis bullosa simplex: implications for disease phenotype and keratin filament assembly. Hum Mutat. 2006;27(7):719-20.

5. Coulombe PA, Hutton ME, Letai A, Hebert A, Paller AS, Fuchs E. Point mutations in human keratin 14 genes of epidermolysis bullosa simplex patients: genetic and functional analyses. Cell. 1991;66(6):1301-11.

6. Takahashi Y, Rouan F, Uitto J, Ishida-Yamamoto A, Iizuka H, Owaribe K, et al. Plectin deficient epidermolysis bullosa simplex with 27-year-history of muscular dystrophy. J Dermatol Sci. 2005;37(2):87-93.

7. Tu WT, Chen PC, Hou PC, Huang HY, Wang JY, Chao SC, et al. Plectin Missense Mutation p.Leu319Pro in the Pathogenesis of Autosomal Recessive Epidermolysis Bullosa Simplex. Acta Derm Venereol. 2020;100(15):adv00242.

8. Hou PC, Natsuga K, Tu WT, Huang HY, Chen B, Chen LY, et al. Complexity of Transcriptional and Translational Interference of Laminin-332 Subunits in Junctional Epidermolysis Bullosa with LAMB3 Mutations. Acta Derm Venereol. 2021;101(8):adv00522.

9. Hung JH, Hou PC, Huang FC, Hsu CK. Topical gentamicin ointment induces LAMB3 nonsense mutation readthrough and improves corneal erosions in a patient with junctional epidermolysis bullosa. Clin Exp Ophthalmol. 2021;49(3):309-12.

10. Hou PC, Tu WT, Chen PC, Guevara BEK, Yen YF, Huang HY, et al. A de novo COL17A1 splice-site mutation causing a 7-bp deletion in a Taiwanese patient with junctional epidermolysis bullosa. Eur J Dermatol. 2021;31(2):267-9.

11. Has C, Kiritsi D, Mellerio JE, Franzke CW, Wedgeworth E, Tantcheva-Poor I, et al. The missense mutation p.R1303Q in type XVII collagen underlies junctional epidermolysis bullosa resembling Kindler syndrome. J Invest Dermatol. 2014;134(3):845-9.

12. D'Alessio M, Zambruno G, Charlesworth A, Lacour JP, Meneguzzi G. Immunofluorescence analysis of villous trophoblasts: a tool for prenatal diagnosis of inherited epidermolysis bullosa with pyloric atresia. J Invest Dermatol. 2008;128(12):2815-9.

13. Pulkkinen L, Cserhalmi-Friedman PB, Tang M, Ryan MC, Uitto J, Christiano AM. Molecular analysis of the human laminin alpha3a chain gene (LAMA3a): a strategy for mutation identification and DNA-based prenatal diagnosis in Herlitz junctional epidermolysis bullosa. Lab Invest. 1998;78(9):1067-76.

14. Varki R, Sadowski S, Uitto J, Pfendner E. Epidermolysis bullosa. II. Type VII collagen mutations and phenotype-genotype correlations in the dystrophic subtypes. J Med Genet. 2007;44(3):181-92.

15. Christiano AM, Morricone A, Paradisi M, Angelo C, Mazzanti C, Cavalieri R, et al. A glycine-to-arginine substitution in the triple-helical domain of type VII collagen in a family with dominant dystrophic epidermolysis bullosa. J Invest Dermatol. 1995;104(3):438-40.

16. Almaani N, Liu L, Dopping-Hepenstal PJ, Lai-Cheong JE, Wong A, Nanda A, et al. Identical glycine substitution mutations in type VII collagen may underlie both dominant and recessive forms of dystrophic epidermolysis bullosa. Acta Derm Venereol. 2011;91(3):262-6.

17. Chao SC, Lee JY. Mutation analyses of COL7A1 gene in three Taiwanese patients with severe recessive dystrophic epidermolysis bullosa. J Formos Med Assoc. 2007 Jan;106(1):86-91.

18. Yu Y, Wang Z, Mi Z, Sun L, Fu X, Yu G, et al. Epidermolysis Bullosa in Chinese Patients: Genetic Analysis and Mutation Landscape in 57 Pedigrees and Sporadic Cases. Acta Derm Venereol. 2021;101(7):adv00503.

19. Wertheim-Tysarowska K, Sobczynska-Tomaszewska A, Kowalewski C, Kutkowska-Kazmierczak A, Wozniak K, Niepokoj K, et al. Novel and recurrent COL7A1 mutation in a Polish population. Eur J Dermatol. 2012;22(1):23-8.

20. Kon A, Pulkkinen L, Ishida-Yamamoto A, Hashimoto I, Uitto J. Novel COL7A1 mutations in dystrophic forms of epidermolysis bullosa. J Invest Dermatol. 1998;111(3):534-7.

21. Rodriguez FA, Gana MJ, Yubero MJ, Zillmann G, Kramer SM, Catalan J, et al. Novel and recurrent COL7A1 mutations in Chilean patients with dystrophic epidermolysis bullosa. J Dermatol Sci. 2012;65(2):149-52.

22. Kern JS, Gruninger G, Imsak R, Muller ML, Schumann H, Kiritsi D, et al. Forty-two novel COL7A1 mutations and the role of a frequent single nucleotide polymorphism in the MMP1 promoter in modulation of disease severity in a large European dystrophic epidermolysis bullosa cohort. Br J Dermatol. 2009;161(5):1089-97.

23. Dunnill MG, Richards AJ, Milana G, Mollica F, Eady RA, Pope FM. A novel homozygous point mutation in the collagen VII gene (COL7A1) in two cousins with recessive dystrophic epidermolysis bullosa. Hum Mol Genet. 1994;3(9):1693-4.

24. Huang L, Wong YP, Burd A. A novel homozygous splice site mutation in COL7A1 in a Chinese patient with severe recessive dystrophic epidermolysis bullosa and squamous cell carcinoma. Int J Dermatol. 2011;50(1):52-6.

25. Christiano AM, LaForgia S, Paller AS, McGuire J, Shimizu H, Uitto J. Prenatal diagnosis for recessive dystrophic epidermolysis bullosa in 10 families by mutation and haplotype analysis in the type VII collagen gene (COL7A1). Mol Med. 1996;2(1):59-76.

26. Shimizu H, McGrath JA, Christiano AM, Nishikawa T, Uitto J. Molecular basis of recessive dystrophic epidermolysis bullosa: genotype/phenotype correlation in a case of moderate clinical severity. J Invest Dermatol. 1996;106(1):119-24.

27. Whittock NV, Ashton GH, Mohammedi R, Mellerio JE, Mathew CG, Abbs SJ, et al. Comparative mutation detection screening of the type VII collagen gene (COL7A1) using the protein truncation test, fluorescent chemical cleavage of mismatch, and conformation sensitive gel electrophoresis. J Invest Dermatol. 1999;113(4):673-86.
