# Supplementary material for: Mutational analysis of epidermolysis bullosa in Taiwan by whole-exome sequencing complemented by RNA sequencing: a series of 77 patients
Source: Orphanet J Rare Dis. 2022 Dec 28;17:451. doi: 10.1186/s13023-022-02605-1 (PMC9795651; doi:10.1186/s13023-022-02605-1)
Supplement: Supplementary file 4 — Additional file 4. Supplementary Table 2. Consequences of splice site mutations. [file 13023_2022_2605_MOESM4_ESM.docx]

**Supplementary table 2. Consequences of splice site mutations**

| Mutation | Novel mutation | Effect on splicing and reading frame | Consequences |
| --- | --- | --- | --- |
| PLEC_c.194-1G>C | Yes | Multiple splicing transcripts downstream to exon 2 | r.? |
| LAMB3_c.373-9T>A† | [1] | Skipping of the entire exon 6 (192 nts) | r.373_564del (p.Gly125_Lys188del) |
| LAMB3_c.629-12T>A† | [2] | Insertion of the last 10 nts of intron 7, leading to frameshift and PTC formation | r.628_629ins629-10_629-1 (p.Glu210GlyfsTer51) |
| LAMB3_ c.2137+1G>T† | [3] | Skipping of the entire exon 15 (161 nts), leading to frameshift and PTC formation | r.1977_2137del (p.Arg660SerfsTer66) |
| COL17A1_c.2003-1G>C† | [4] | Deletion of the first 7 nts of exon 25, leading to frameshift and PTC formation | r.2003_2009del (p.Gly668AlafsTer13) |
| COL7A1_c.5499C>T† | [5] | Deletion of the 11th to the last nt of exon 64 (35 nts), leading to frameshift and PTC formation | r.5498_5532del (p.Glu1834ArgfsTer26) |
| COL7A1_c.5532+5G>A† | [6] | Skipping of the entire exon 64 (45 nts) | r.5488_5532del (p.Gly1830_Asn1844del) |
| COL7A1_c.5820+4A>G‡ | Yes | Major transcript: Retention of the intron 70 (346 nts), minor transcript: skipping of exon 70 (48 nts) | r.[5820_5821ins5820+1_5820+346, 5773_5820del] (p.[Pro5820_Asn5821ins30, Gly1925_Pro1940del]) |
| COL7A1_c.6394-1G>A | Yes | Deletion of the first nt of exon 78, leading to frameshift and PTC formation | r.6394del (p.Gly2132ValfsTer74) |
| COL7A1_c.6501G>A† | [7] | Insertion of the first 49 nts of intron 79, leading to frameshift and PTC formation | r.6501_6502ins6501+1_6501+49 (p.Gly2168ValfsTer5) |
| COL7A1_c.7272G>A | Yes | Skipping of both exon 93 (60 nts) and exon 94 (108 nts) | r.7105_7272del (p.Gly2369_Arg2424del) |
| COL7A1_c.8304+5G>A | Yes | Skipping of the entire exon 111 (78 nts) | r.8227_8304del (p.Gly2743_Gln2768del) |
| COL7A1_c.8407+5G>C* | [8] | Skipping of the entire exon 113 (49 nts), leading to frameshift and PTC formation | r.8359_8407del (p.Thr2787ProfsTer95) |

nt(s): nucleotide(s)

*These mutations have been reported before, but its consequences were not investigated.

†These mutations, including their consequences, have been reported before.

‡c.5820+4A>G leads to multiple transcripts. r.5820_5821ins5820+1_5820+346 (retention of intron 70, as shown by RNA sequencing) is a major transcript, and r.5773_5820del (skipping of exon 70, as shown by RT-PCR and Sanger sequencing) is a minor transcript.

***References***

1. Hung JH, Hou PC, Huang FC, Hsu CK. Topical gentamicin ointment induces LAMB3 nonsense mutation readthrough and improves corneal erosions in a patient with junctional epidermolysis bullosa. Clin Exp Ophthalmol. 2021;49(3):309-12.

2. Chen F, Huang L, Li C, Zhang J, Yang W, Zhang B, et al. Next-generation sequencing through multigene panel testing for the diagnosis of hereditary epidermolysis bullosa in Chinese population. Clin Genet. 2020;98(2):179-84.

3. Hou PC, Natsuga K, Tu WT, Huang HY, Chen B, Chen LY, et al. Complexity of Transcriptional and Translational Interference of Laminin-332 Subunits in Junctional Epidermolysis Bullosa with LAMB3 Mutations. Acta Derm Venereol. 2021;101(8):adv00522.

4. Has C, Kiritsi D, Mellerio JE, Franzke CW, Wedgeworth E, Tantcheva-Poor I, et al. The missense mutation p.R1303Q in type XVII collagen underlies junctional epidermolysis bullosa resembling Kindler syndrome. J Invest Dermatol. 2014;134(3):845-9.

5. Kern JS, Gruninger G, Imsak R, Muller ML, Schumann H, Kiritsi D, et al. Forty-two novel COL7A1 mutations and the role of a frequent single nucleotide polymorphism in the MMP1 promoter in modulation of disease severity in a large European dystrophic epidermolysis bullosa cohort. Br J Dermatol. 2009;161(5):1089-97.

6. Huang L, Wong YP, Burd A. A novel homozygous splice site mutation in COL7A1 in a Chinese patient with severe recessive dystrophic epidermolysis bullosa and squamous cell carcinoma. Int J Dermatol. 2011;50(1):52-6.

7. Christiano AM, LaForgia S, Paller AS, McGuire J, Shimizu H, Uitto J. Prenatal diagnosis for recessive dystrophic epidermolysis bullosa in 10 families by mutation and haplotype analysis in the type VII collagen gene (COL7A1). Mol Med. 1996;2(1):59-76.

8. Chao SC, Lee JY. Mutation analyses of COL7A1 gene in three Taiwanese patients with severe recessive dystrophic epidermolysis bullosa. J Formos Med Assoc. 2007;106(1):86-91.
