# Supplementary material for: Mutational analysis of epidermolysis bullosa in Taiwan by whole-exome sequencing complemented by RNA sequencing: a series of 77 patients
Source: Orphanet J Rare Dis. 2022 Dec 28;17:451. doi: 10.1186/s13023-022-02605-1 (PMC9795651; doi:10.1186/s13023-022-02605-1)
Supplement: Supplementary file 5 — Additional file 5. Supplementary Table 3. Genotype and clinical presentation of patients with EB pruriginosa. [file 13023_2022_2605_MOESM5_ESM.docx]

**Supplementary Table 3. Genotype and clinical presentation of patients with EB pruriginosa**

| Family | Patient | Mode of inheritance | Genotype | Nail dystrophy* | Distribution of prurigo-like lesions | Novelty | Other EB patients in the family | Reported phenotypes in representative literature |
| --- | --- | --- | --- | --- | --- | --- | --- | --- |
| 23 | 34 | AD | c.6127G>A (p.Gly2043Arg) (Het) | Toenails (10)  Fingernails (7) | Numerous confluent nodules and plaques localized to the elbows, dorsal hands, knees, lower legs | [1] | AD-DEB, localized (PT33) | AD-DEB-localized [2, 3] |
| 29 | 51 | AD |  | Toenails (10)  Fingernails (8) | Numerous confluent nodules and plaques on most part of the trunk, the upper and lower extremities |  | AD-DEB, intermediate (PT52) |  |
| 24 | 37 | AD | c.7697G>A (p.Gly2566Glu) (Het) | Toenails (10)  Fingernails (10) | Numerous confluent nodules and plaques on some area of the lower extremities | Novel | AD-DEB, localized (PT35, PT36) | No previous reports |
| 25 | 40 | AD | c.6182G>A (p.Gly2061Glu) (Het) | Toenails (10)  Fingernails (10) | Numerous confluent nodules and plaques on most part of the trunk, the upper and lower extremities | [4] | AD-DEB, localized (PT38, PT39) | AR-DEB-severe [4] |
| 27 | 43 | AD | c.4670G>A (p.Gly1557Glu) (Het) | Toenails (0)  Fingernails (0) | Scattered nodules on the trunk, the upper and lower extremities | [5] | AD-DEB, localized (PT42) | AD-DEB-localized [5, 6] |
|  | 44 |  |  | Toenails (no data)  Fingernails (0) | Numerous nodules on some area of the forehead, upper and lower extremities |  |  |  |
|  | 45 |  |  | Toenails (3)  Fingernails (no data) | Numerous nodules localized to the lower legs |  |  |  |
|  | 46 |  |  | Toenails (10)  Fingernails (1) | Numerous nodules localized to the lower legs, knees, upper arms, and elbows |  |  |  |
|  | 47 |  |  | Toenails (0)  Fingernails (0) | Numerous nodules and papules on some area of the trunk, the upper and lower extremities |  |  |  |
|  | 48 |  |  | Toenails (5)  Fingernails (0) | Some papules and nodules on certain area of the lower legs, elbows, and the trunk |  |  |  |
| 28 | 49 | AD | c.5318G>T (p.Gly1773Val) (Het) | Toenails (5)  Fingernails (0) | Some papules and nodules on certain area of the upper extremities, as well as confluent nodule and plaques on the lower legs | [2] | No other EB patients in this family | AD-DEB-localized [2] AD-DEB-pruriginosa [7] |
|  | 50 |  |  | Toenails (10)  Fingernails (2) | Confluent nodules and plaques localized to the lower legs |  |  |  |
| 30 | 57 | AD |  | Toenails (no data)  Fingernails (no data) | Confluent nodules and plaques localized to the lower legs |  | AD-DEB, localized (PT53, PT54, PT55) AD-DEB, intermediate (PT56) |  |
|  | 58 |  |  | Toenails (10)  Fingernails (7) | Confluent nodules and plaques localized to the lower legs |  |  |  |
| 26 | 41 | AR | c.5820+4A>G (Het) c.3562G>A (p.Val1188Met) (Het) | Toenails (10)  Fingernails (0) | Confluent nodules localized to the lower legs and dorsal feet | Novel Novel | No other EB patients in this family | No previous reports |

* Numbers in brackets indicate the number of affected toenails and fingernails, respectively.

**References**

1. Christiano AM, Morricone A, Paradisi M, Angelo C, Mazzanti C, Cavalieri R, et al. A glycine-to-arginine substitution in the triple-helical domain of type VII collagen in a family with dominant dystrophic epidermolysis bullosa. J Investig Dermatol. 1995;104(3):438-40.

2. Wertheim-Tysarowska K, Sobczynska-Tomaszewska A, Kowalewski C, Kutkowska-Kazmierczak A, Wozniak K, Niepokoj K, et al. Novel and recurrent COL7A1 mutation in a Polish population. Eur J Dermatol. 2012;22(1):23-8.

3. Cserhalmi-Friedman PB, Karpati S, Horvath A, Christiano AM. Identification of the glycine-to-arginine substitution G2043R in type VII collagen in a family with dominant dystrophic epidermolysis bullosa from Hungary. Exp Dermatol. 1997;6(6):303-7.

4. Chao SC, Lee JY. Mutation analyses of COL7A1 gene in three Taiwanese patients with severe recessive dystrophic epidermolysis bullosa. J Formos Med Assoc. 2007;106(1):86-91.

5. Christiano AM, McGrath JA, Tan KC, Uitto J. Glycine substitutions in the triple-helical region of type VII collagen result in a spectrum of dystrophic epidermolysis bullosa phenotypes and patterns of inheritance. Am J Hum Genet. 1996;58(4):671-81.

6. Yu Y, Wang Z, Mi Z, Sun L, Fu X, Yu G, et al. Epidermolysis Bullosa in Chinese Patients: Genetic Analysis and Mutation Landscape in 57 Pedigrees and Sporadic Cases. Acta Derm Venereol. 2021;101(7):adv00503.

7. Tang ZL, Lin ZM, Wang HJ, Chen Q, Xu XM, Ge HF, et al. Four novel and two recurrent glycine substitution mutations in the COL7A1 gene in Chinese patients with epidermolysis bullosa pruriginosa. Clin Exp Dermatol. 2013;38(2):197-9.
